# Social Media Overload as a Predictor of Depressive Symptoms Under the COVID-19 Infodemic: A Cross-Sectional Survey From Chinese University Students

**DOI:** 10.3389/ijph.2023.1606404

**Published:** 2023-10-20

**Authors:** Tian Xie, Yangyang Wang, Yali Cheng

**Affiliations:** ^1^ School of Media and Communication, Shanghai Jiao Tong University, Shanghai, China; ^2^ China Institute for Urban Governance, Shanghai Jiao Tong University, Shanghai, China; ^3^ School of International and Public Affairs, Shanghai Jiao Tong University, Shanghai, China; ^4^ School of Journalism, Fudan University, Shanghai, China

**Keywords:** social media overload, depressive symptoms, public health, risk perception, social media fatigue

## Abstract

**Objectives:** People’s mental health and digital usage have attracted widespread attention during the COVID-19 pandemic. This study aimed to investigate how social media overload influenced depressive symptoms under the COVID-19 infodemic and the role of risk perception and social media fatigue.

**Methods:** A questionnaire survey was conducted on 644 college students during the COVID-19 lockdown in Shanghai, and data analysis was conducted using the PROCESS4.0 tool.

**Results:** The findings showed that in the COVID-19 information epidemic: 1) both information overload and communication overload were significantly and positively associated with depressive symptoms; 2) risk perception of COVID-19, and social media fatigue mediated this association separately; 3) and there was a chain mediating relationship between communication overload and depressive symptoms.

**Conclusion:** Social media overload was positively associated with depressive symptoms among college students under the COVID-19 infodemic by increasing risk perception and social media fatigue. The findings sparked further thinking on how the public should correctly use social media for risk communication during public health emergencies.

## Introduction

COVID-19 has been regarded as the first pandemic in the digital age [1]. According to the World Health Organization (WHO), healthcare institutions are battling not only epidemics but also the infodemic. In the early stages of the Omicron wave, Shanghai experienced a citywide lockdown, during which rumors and misinformation quickly spread through social media. Compared to traditional media, social media are frequently seen as being more interactive, actual, credible, and dialogic [2]. Through social media, epidemic information spreads faster and more widely. Since the outbreak of the COVID-19 pandemic, people’s use of social media has increased significantly [3]. People rely heavily on social media to get information and connect with others. Social media reflects people’s attention to public health emergencies [4]. The role of the media expected by society is to provide accurate and timely information about epidemics so that citizens can respond promptly [5]. However, social media is not only a platform for information transmission and risk communication but also an amplification station for overloaded information and communication. The concept of social overload was first proposed to describe the negative effects of population congestion [6]. According to the bounded rationality theory, Social media overload refers to an individual’s social media processing capacity falling short of the massive information and social input [7].

A systematic review pointed out that four aspects of social media including time spent on, activity, investment, and addiction are associated with negative mental health outcomes such as depression, anxiety, and stress [8]. Research showed that the popularity of COVID-19 has affected people’s mental health, the prevalence of depressive symptoms during the COVID-19 pandemic was more than three times higher than before the pandemic [9]. On the Chinese search engine Baidu, there is an upsurge in inquiries about depression [10]. Besides, rates of depression among college students are significantly greater than those in the general population [11]. A systematic review found that the prevalence of depressive symptoms was 34% among higher education students, which was higher compared to pre-pandemic prevalence in similar populations [12]. It is necessary to explore the predictors of depressive symptoms and their influence mechanism among college students during the pandemic lockdown.

This study explores the potential mechanism of the impact of social media overload on depression symptoms among college students in the context of the infodemic. The present study has three purposes: 1) to provide a new perspective on the influencing factors of college students’ mental health. 2) Examine the underlying mechanism of how social media overload affects depressive symptoms during the lockdown. 3) Explore further strategies for the proper use of social media during a pandemic to avoid negative effects on mental health.

### Social Media Overload and Depressive Symptoms

Social media which is built on the philosophical and technical principles of Web 2.0 are defined as internet applications that enable the production and distribution of user-generated content [13]. There are two primary domains of social media overload: information overload (IO) and communication overload (CO). IO refers to an individual’s information processing ability that cannot meet massive information inputs at a high rate [14, 15]. The information redundancy and low quality of information brought by social media cause people to spend a lot of time processing information, which brings negative emotions [16, 17]. Previous studies pointed out that COVID-19 information overload positively affects depressive symptoms [18, 19]. CO is defined as a situation when communication demands from diverse sources exceed an individual’s communication capacity [20, 21]. Social media is used as a means of rapid communication in a crisis [16]. One study proved that interpersonal communication about COVID-19 had no significant effect on depression [17]. However, the means of social media communication include not only person-to-person communication but also group communication and mass communication [22]. Overloaded communication about the pandemic may magnify the severity of the pandemic, and increase worry and stress, which may result in depressive symptoms. Reinecke et al. pointed out that communication overload had a significant indirect effect on depression through perceived stress [23]. However, according to a survey of Romanian social media users, excessive COVID-19-related information had no significant effect on depression during the lockdown [1]. Therefore, the association between social media overload and depressive symptoms is still to be confirmed, we hypothesized the following:

H1a Information overload will be positively associated with depressive symptoms

H1b Communication overload will be positively associated with depressive symptoms

### The Mediating Role of Risk Perception of COVID-19

Risk perception refers to people’s subjective judgments and evaluations of hazards that they are or may experience [24, 25]. Social media plays an irreplaceable role in shaping people’s risk perceptions [26]. It has huge potential in forming an individual’s perception of risk, conceptions, fears, and opinions collide in social media interactions [27]. Kasperson et al put forward the “social amplification of risk” framework, considering social media as an amplification for perception of risk [25]. High social media exposure was substantially linked to greater levels of COVID-19 risk perception, anxiety, and depression [28]. It has been proved that information overload was positively related to perceived risk of the vaccine [27], and colon cancer risk perceptions [29]. Students with high levels of information-seeking reported a greater risk perception of COVID-19 [30]. Dryhurst suggested that social amplification through friends and families were important determinants of risk perception [31]. During the COVID-19 lockdown, online interaction with family and friends through social media has been the main channel of communication. Hence, information overload and communication overload may have a positive effect on the risk perception of COVID-19. Besides, risk perception has been widely examined as a predictor of depression [32, 33]. One study showed that affective risk perception had a positive effect on depression, while cognitive risk perception has a negative effect on depression [34]. Hence, we propose the following hypotheses:

H2a Risk perception of COVID-19 mediates the relationship between information overload and depressive symptoms

H2b Risk perception of COVID-19 mediates the relationship between communication overload and depressive symptoms

### The Mediating Role of Social Media Fatigue

Social media fatigue (SMF) is defined as negative emotions such as exhaustion and tiredness caused by social media use, and the tendency to withdraw from social media [21, 35]. Individuals may feel overwhelmed, fatigued, and anxious when faced with massive information and communication [7, 36]. The stressor-strain-outcome (SSO) model is the main theoretical framework to explain social media fatigue. This model reveals how social media overload affects strain variables. Based on the SSO model, studies found that information overload and communication overload were significant stressors of social media fatigue [37, 38]. A study in the context of the pandemic also suggested that information overload significantly affects emotional exhaustion [39]. However, one study holds a different opinion that communication overload had no significant effect on fatigue [40]. In addition, social media fatigue is an important predictor of negative psychological health outcomes, such as anxiety, stress, and depression [37, 41]. COVID-19-related fatigue was found to predict depression in a significant and beneficial way [42]. It has also been found that social media fatigue mediated the association between information overload and social anxiety [43]. Therefore, we proposed the following assumptions:

H3a Social media fatigue (SMF) mediates the relationship between information overload and depressive symptoms

H3b Social media fatigue (SMF) mediates the relationship between communication overload and depressive symptoms

### The Chain Mediating Model

Perceived risk of COVID-19 was negatively related to mental health, and emotions mediated this relationship [44]. Risk perception of SNS may increase social network fatigue [45]. In the context of the COVID-19 pandemic, some studies explored the relationship between risk perception and fatigue. One study showed that the effect of fatigue on protective behaviors reduced as the perceived risk of COVID-19 increased [46]. Another study pointed out that pandemic risk perception can be also a predictor of pandemic fatigue through the mediation of perceived stress and preventive coping [47]. Different kinds of fatigue have been proven to be associated with risk perception. Employee fatigue in healthcare settings has been the subject of numerous types of research. Job-related risks increase employees’ susceptibility to exhaustion [48]. Social media amplifies the level of risk of COVID-19, and when people perceive more risk, they will become tired and reluctant to social media. Therefore, we proposed the following assumptions:

H4a Information overload will be positively associated with depressive symptoms through the chain mediating role of risk perception of COVID-19 and social media fatigue.

H4b Communication overload will be positively associated with depressive symptoms through the chain mediating role of risk perception of COVID-19 and social media fatigue.

**FIGURE 1:**
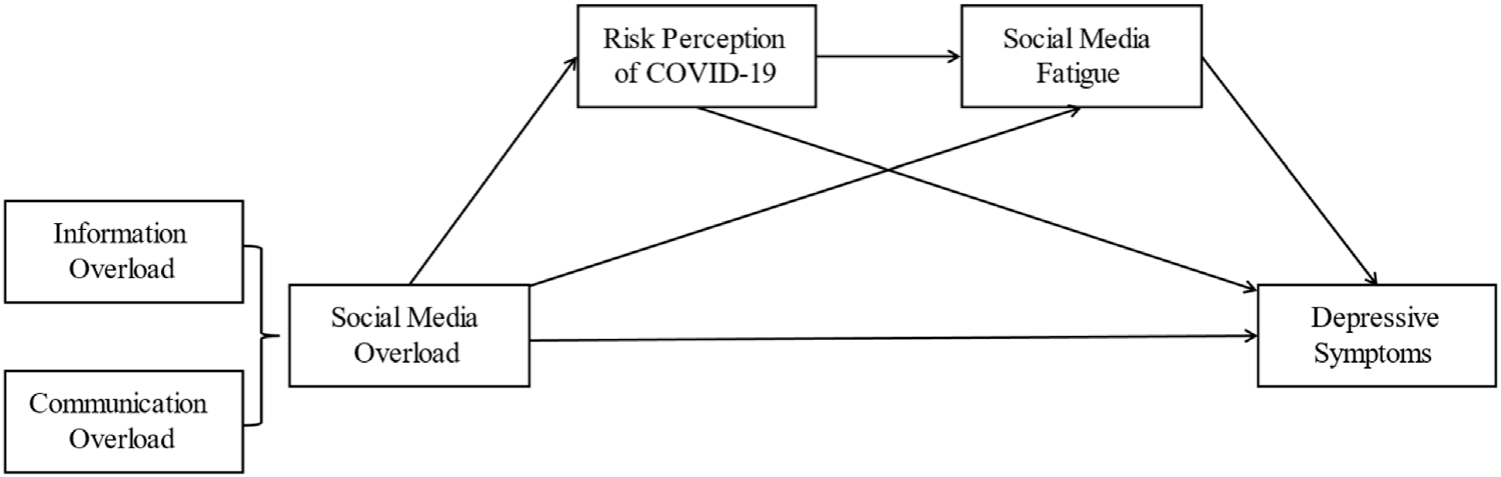
Theoretical model (Shanghai, China. 2022).

Figure 1 shows the theoretical model of the relationship between social media overload and depressive symptoms.

## Methods

### Sample and procedure

Between the 25th of March and the 13th of April 2022, we surveyed a group of university students enrolled in Shanghai using Wen Juan Xing (www.wjx.cn), the largest online survey platform in China, which is the equivalent of Qualtrics and SurveyMonkey, providing online data for business and academic fields [49–53]. Its sample database contains over 2.6 million respondents who confirmed their personal information [52], and more than 10 million people on average fill out questionnaires on the platform every day, reaching nearly 300 million users every month, and accurate questionnaires can be placed according to user interest labels and specific groups now. Our survey was conducted during the COVID-19 pandemic and the Omicron Wave Lockdown in Shanghai. The specific steps were that the Wen Juan Xing survey platform first randomly selected respondents who met the requirements for interview and data collection in its huge sample pool and then invited this group of respondents to find new potential respondents to answer the questionnaire through social media and other means, and in this way circulated the respondents who forwarded the diffusion questionnaire until the number of questionnaires recovered reached 800. The average response time per student was 810.25 s, and after excluding invalid samples based on response time and basic information, and further controlling the sample with quotas based on age, gender, and education information, 644 valid samples were finally retained.

### Respondents’ Information

The total sample included 383 female and 261 male college students, with a larger proportion of female (59.5%) respondents than male (40.5%). The proportion of respondents under the age of 20 was 18.3%, and the largest proportion were in the 20–21 (30.4%) and 22–23 (30.1%) age groups, but the smallest proportion was in the 30 and above (0.3%) age group. In terms of the academic stage, the largest number of respondents were undergraduate students (80.4%), followed by master’s students (14.9%) and doctoral students (4.6%). 63.2% of respondents had a campus outbreak at their school, 64.3% lived in the school dormitory, but only 1.1% of respondents had COVID-19 negative. In addition, during the Shanghai Omicron Wave Lockdown period, most respondents spent 1–3 h (45.3%) and 4–5 h (28.4%) per day on social media, but 12.7% of respondents spent more than 5 h per day on social media and 13.5% spent less than 1 h per day or did not use it.

### Measures

#### Information Overload (IO)

Information overload was assessed by adapting scales from Zhang et al.’s study [54]. The scale contains four items and was measured using a five-degree Likert scale (1 = strongly disagree, 5 = strongly agree). The results of respondents’ responses were aggregated and the mean was calculated to measure the extent of information overload. Its Cronbach’s α is 0.832, and five items had factor loadings values of 0.535, 0.725, 0.765, 0.779, and 0.709 for the confirmatory factor analysis (CFA).

#### Communication Overload (CO)

It was measured concerning Cho et al.’s study [55, 56]. The scale contains four items and was measured using a five-degree Likert scale (1 = strongly disagree, 5 = strongly agree). Four five-point Likert scale items (1 = strongly disagree, 5 = strongly agree) were used to measure respondents’ level of agreement. The results of respondents’ replies were aggregated and the mean was calculated to measure the degree of communication overload. Its Cronbach’s α is 0.668, and the factor loadings values of CFA for the four items were 0.628, 0.636, 0.569, and 0.568.

#### Risk Perception of COVID-19 (RPC)

Risk Perception of COVID-19 was measured based on the scale developed by Zhuang et al. [57] Four five-point Likert scale items (1 = strongly disagree, 5 = strongly agree) were used to measure risk perception of COVID-19. Respondents’ responses were summarized and the mean was calculated to measure the extent of risk perception of COVID-19. Its Cronbach’s α is 0.795, and four items had CFA factor loadings values of 0.582, 0.531, 0.840, and 0.840.

#### Social Media Fatigue (SMF)

Social Media Fatigue was measured from a study by Maier et al. [58]. Three five-point Likert scale items (1 = strongly disagree, 5 = strongly agree) were used to measure social media fatigue. Respondents’ responses were totaled and the mean was calculated to measure the extent of social media fatigue. Its Cronbach’s α is 0.872, and three items had CFA factor loading values that were 0.815, 0.882, and 0.806, respectively.

#### Depressive Symptoms (DS)

Depressive symptoms were measured on a scale from the study by Salokangas et al. [59]. Five 5-point Likert scale items (1 = strongly disagree, 5 = strongly agree) were used. This measurement has been used in prior research [60, 61]. Respondents’ answers were pooled and the mean was calculated to construct the depressive symptoms measure score. Its Cronbach’s α is 0.749, and the CFA factor loadings values for the five items were 0.587, 0.510, 0.704, 0.578, and 0.741.

All the measurements are listed in Appendix A. In addition, the variable measures section above reports their respective structural validity and factor loading values for each question item, indicating that the requirements for further analysis were met. The adjusted CFA model has good fit indicators, where χ^2^/df is 2.172 (it is generally accepted that less than 5 is acceptable and less than 3 is good); IFI, TLI, and CFI are all greater than 0.9; PGFI, PNFI, and PCFI are greater than 0.7, 0.7, and 0.8, 0.9, respectively; and RMSEA is less than 0.05.

#### Control Variable

Three kinds of control variables were selected including socio-demographic characteristics, school characteristics, and life status. Specifically, the sociodemographic characteristics variables included gender (Female, Male), age, and university stage (Bachelor’s degree in progress, Master’s degree in progress, Doctorate in progress). School characteristics included school outbreaks (Yes, No), and university type (General University; Key University; Top University). Life state characteristic variables included on-campus accommodation (Yes, No). COVID-19 negative (Yes; No), residence status (Living alone; Others), and social media usage (Never use; <1 h; 1∼3 h; 4∼5 h; 5 h <).

### Statistical Analysis

With the help of SPSS26, we did the statistical analysis and common method bias for all variables and Pearson correlation analysis for the main variables. Mediated effects analysis can help the study identify the mechanism of action by which social media overload exerts its influence on depression, as well as the calculation and comparison of total, direct, and indirect effects. In testing the multiple mediating effects, we were conducted using model 6 of the macro PROCESS4.0 tool [62], a tool that helps us to estimate the multiple mediating effects model, as well as the total, direct, and indirect effects of the model [63]. We used 5,000 bootstrap samples randomly selected from the sample to calculate indirect effects and confidence intervals. In particular, it should be noted that in bootstrap analysis, the impact effect of this path is considered significant when the confidence interval does not include 0.

### Common Method Bias

Common method bias, which is artificial covariance among measurement variables due to the same data source or respondents, the same measurement environment, the item discourse, and the characteristics of the items themselves, can lead to biased research findings. Harman’s single-factor test is a diagnostic technique for evaluating the severity of common method bias [64]. We conducted Harman’s single-factor test using SPSS26 for the main variables measured. The results showed that the variance of the first single factor was 29.523%, which is below the limit of 40%. This indicates that the quality of the data is adequate for further analysis.

## Results

### Correlation Analysis Between Main Variables

Bivariate correlations were conducted for the main variables using Pearson correlation, as well as their means and variances were calculated (see Table 1). Positive correlations were found between depressive symptoms, information overload, communication overload, risk perception of COVID-19, and social media fatigue, all of which were statistically significant at the 1% level of significance. Specifically, information overload (*r* = 0.326, *p* < 0.01) and communication overload (*r* = 0.219, *p* < 0.001) were both positively correlated with depressive symptoms, indicating that college students with higher levels of information overload and communication overload also had higher rates of depressive symptoms during the omicron lockdown. information overload (*r* = 0.503, *p* < 0.01) and communication overload (*r* = 0.206, *p* < 0.01) were both positively correlated with risk perception of COVID-19, and both information overload (*r* = 0.366, *p* < 0.01) and communication overload (*r* = 0.301, *p* < 0.01) were also positively correlated with social media fatigue, which indicates that college students with higher levels of social media overload also had higher perceived risk of COVID-19 and social media burnout. In addition, risk perception of COVID-19 and social media fatigue were positively correlated (*r* = 0.191, *p* < 0.01), and both risk perception of COVID-19 (*r* = 0.318, *p* < 0.01) and social media fatigue (*r* = 0.357, *p* < 0.01) were also significantly positively correlated with depressive symptoms, suggesting that college students with higher perceived risk during omicron lockdown also had higher levels of social media fatigue, as well as a higher prevalence of depressive symptoms. The plausibility of the proposed research hypothesis was initially confirmed.

**TABLE 1:** Correlation analysis between main variables (*N* = 644) (Shanghai, China. 2022).

Variables	Mean	SD	DS	IO	CO	RPC	SMF
Depressive symptoms (DS)	2.491	0.797	—				
Information Overload (IO)	3.277	0.977	0.326***	—			
Communication Overload (CO)	2.980	0.925	0.219***	0.364***	—		
Risk Perception of COVID-19 (RPC)	3.746	0.919	0.318***	0.503***	0.206***	—	
Social Media Fatigue (SMF)	2.598	1.039	0.357***	0.366***	0.301***	0.191***	—

Note: ***p < 0.01.

### The Chain Mediating Effect of Information Overload and Depressive Symptoms

**FIGURE 2:**
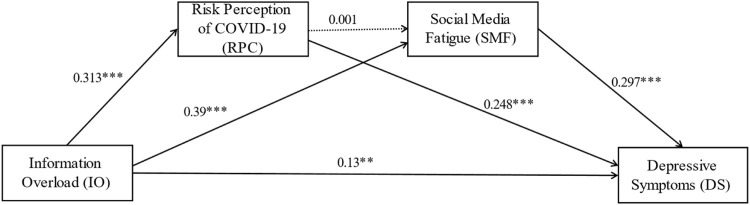
The chain mediating effect of information overload and depressive symptoms (Shanghai, China. 2022). Note: Unstandardized path coefficients. The dotted line indicates that the path coefficients are not statistically significant. **p < 0.01; ***p < 0.001.

After controlling for the three types of control variables, we tested the hypotheses using model 6 of SPSS PROCESS 4.0 macro, and the results are shown in Figure 2 and Table 2. Specifically, the “Total effect” in Table 2 indicates that information overload has a positive relationship with depressive symptoms (*b* = 0.323, CI = 0.259–0.388), which supports hypothesis H1a: Information overload will be positively associated with depressive symptoms.

**TABLE 2:** Total effect, direct effect, and indirect effect of the multiple mediating effects (Shanghai, China. 2022).

Effect or indirect path	Effect	Boot SE	Boot LLCI	Boot ULCI	Ratio of indirect to total effect	Ratio of indirect to direct effect
Total effect	0.323	0.033	0.259	0.388	—	—
Direct effect	0.130	0.040	0.052	0.208	—	—
Indirect effect	0.194	0.028	0.139	0.249	60.06%	149.23%
IO -> RPC -> DS	0.078	0.017	0.046	0.112	24.15%	60.00%
IO -> SMF -> DS	0.116	0.022	0.073	0.159	35.91%	89.23%

Note: Boot SE, Boot LLCI, and Boot ULCL, are estimated standard error under bias-corrected percentile bootstrap method, and 95% confidence interval lower and 95% confidence interval upper, and Boot LLCI, and Boot ULCL, do not overlap with zero, number of bootstrap samples for percentile bootstrap confidence intervals is 5,000.

Figure 2 reports the unstandardized path coefficients of the relationship between information overload, risk perception of COVID-19, and social media fatigue and depressive symptoms. Information overload positively influenced risk perception of COVID-19 (*b* = 0.313, *p* < 0.001), and also Risk Perception of COVID-19 positively influenced Depressive symptoms (*b* = 0.248, *p* < 0.001), which indicated that risk perception of COVID-19 mediated the relationship between information overload and depressive symptoms, and supported hypothesis H2a: Risk Perception of COVID-19 mediates the relationship between information overload and depressive symptoms.

Information Overload positively influenced social media fatigue (*b* = 0.39, *p* < 0.001), and at the same time, social media fatigue positively influenced depressive symptoms (*b* = 0.297, *p* < 0.001), indicating that social media fatigue mediated the relationship between information overload and depressive symptoms, and supported hypothesis H3a: Social media fatigue (SMF) mediates the relationship between information overload and depressive symptoms. In addition, there is no significant correlation between risk perception of COVID-19 and social media fatigue, suggesting the absence of a chain mediating mechanism. Therefore, hypothesis H4a “Information overload will be positively associated with depressive symptoms through the chain mediating role of risk perception of COVID-19 and social media fatigue” was not supported. In terms of control variables, only gender and social media usage had a significant positive effect on depressive symptoms, while the other control variables were not statistically significant.

After verifying the multiple mediating mechanisms of risk perception of COVID-19 and social media fatigue, we proceeded to calculate the total effect, direct effect, and indirect effect of the two mediating paths (Table 3). Specifically, in the process of information overload positively affecting depressive symptoms, the total effect was 0.323, the direct effect was 0.13, and the indirect effect (0.194) accounted for 60.06% and 149.23% of the total and direct effects, respectively. In other words, the proportion of the indirect effect exceeded the direct effect, and this indirect effect played its effect from two mediated paths (a) CO -> RPC -> DS and (b) CO -> SMF -> DS, which accounted for 24.15% and 35.91% of the total effect, respectively. This indicates that the mediating effect of social media fatigue is stronger than the risk perception of COVID-19. In addition, the above tests for total effect, direct effect, and mediating effects (a) and (b), do not include 0 in the 95% confidence interval and are statistically significant.

**TABLE 3:** Total effect, direct effect, and indirect effect of the chain mediating effects (Shanghai, China. 2022).

Effect or indirect path	Effect	Boot SE	Boot LLCI	Boot ULCI	Ratio of indirect to total effect	Ratio of indirect to direct effect
Total effect	0.249	0.047	0.158	0.341	—	—
Direct effect	0.067	0.046	−0.023	0.156	—	—
Indirect effect	0.183	0.024	0.136	0.230	73.49%	273.13%
CO -> RPC -> DS	0.079	0.017	0.047	0.114	31.73%	117.91%
CO -> SMF -> DS	0.090	0.018	0.056	0.128	36.14%	134.33%
CO -> RPC->SMF ->DS	0.014	0.005	0.005	0.024	5.62%	20.90

Note: Boot SE, Boot LLCI, and Boot ULCL, are estimated standard error under bias-corrected percentile bootstrap method, and 95% confidence interval lower and 95% confidence interval upper, and Boot LLCI, and Boot ULCL, do not overlap with zero, number of bootstrap samples for percentile bootstrap confidence intervals is 5,000.

### The Chain Mediating Effect of Communication Overload and Depressive Symptoms

**FIGURE 3:**
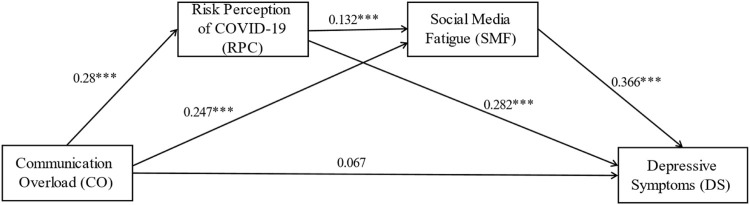
The chain mediating effect of communication overload and depressive symptoms (Shanghai, China. 2022). Note: Unstandardized path coefficients. ***p < 0.001.

After adding the control variables, we proceeded to test the hypotheses using Model 6 of SPSS PROCESS 4.0 macro, and the results are shown in Figure 3 and Table 3. Table 3 in the “Total effect” section shows that communication overload has a positive relationship with depressive symptoms (*b* = 0.249, CI = 0.158–0.341), which supports hypothesis H1b: Communication overload will be positively associated with depressive symptoms.

We reported the unstandardized path coefficients of the relationship between communication overload, risk perception of COVID-19, social media fatigue, and depressive symptoms in Figure 3. Communication overload positively influenced risk perception of COVID-19 (*b* = 0.28, *p* < 0.001), and at the same time, risk perception of COVID-19 positively influenced depressive symptoms (*b* = 0.282, *p* < 0.001), which indicated that risk perception of COVID-19 mediated the relationship between communication overload and depressive symptoms, and supported hypothesis H2b: Risk perception of COVID-19 mediates the relationship between communication overload and depressive symptoms.

Communication overload positively influenced social media fatigue (*b* = 0.247, *p* < 0.001), and meanwhile, social media fatigue positively influenced depressive symptoms (*b* = 0.366, *p* < 0.001), suggesting that social media fatigue mediated the relationship between information overload and depressive symptoms, supporting hypothesis H3b: Social media fatigue (SMF) mediates the relationship between communication overload and depressive symptoms. In addition, risk perception of COVID-19 positively influenced social media fatigue (*b* = 0.132, *p* < 0.001), and then, risk perception of COVID-19 and social media fatigue mediated the relationship between communication overload and depressive symptoms, respectively. Thus, the existence of the chain mediating mechanism was demonstrated, which subsequently supported hypothesis H4b: Communication overload will be positively associated with depressive symptoms through the chain mediating role of risk perception of COVID-19 and social media fatigue. In addition, only gender and social media usage had a significant positive effect on depressive symptoms; the other control variables were not statistically significant.

The same analysis process as in the above section. Following the verification of the chain mediation mechanism of risk perception of COVID-19 and social media fatigue, we proceeded to calculate the total effect, direct effect, and indirect effect for the three mediated paths (Table 3). Specifically, during the process of communication overload positively affecting depressive symptoms, the total effect was 0.249, the direct effect was 0.067, and the indirect effect (0.183) accounted for 73.49% and 273.13% of the total and direct effects, respectively. It indicates that the proportion of indirect effect surpasses the direct effect, and the indirect effect works from three mediated paths of (a) CO -> RPC -> DS, (b) CO -> SMF -> DS and (c) CO -> RPC -> SMF -> DS, which account for 31.73%, 36.14% and 5.62% of the total effect, correspondingly. It shows that the mediating effect of social media fatigue is stronger than the risk perception of COVID-19 and chain mediating paths, and the chain mediating path has the weakest mediating effect. Furthermore, all of the above tests for total effect, direct effect, and mediated effect (a), (b), and (c), did not include 0 in the 95% confidence interval and were statistically significant.

## Discussion

This study examined the association between social media overload and depressive symptoms among university students during the COVID-19 infodemic. The results indicated that information overload and communication overload were positively associated with depressive symptoms, supporting H1a and H1b. The more information and communication people perceive on social media, the higher level of depression they will feel [35]. University students who relied on social media for updates reported being exposed to more contradicting information [65]. An alarming amount of fake information, conspiracy theories, miraculous treatments, and hateful news are circulated during the pandemic [66]. It is difficult to distinguish between true and false, people have to spend time and energy dealing with the overloaded COVID-19-related information and communication, which may lead to depressive symptoms. This finding shows that avoiding social media overload is an effective way to resist infodemic and negative psychological problems.

Risk perception of COVID-19 mediated the relationship between social media overload and depressive symptoms, lending support to hypotheses H2a and H2b. The perception of public risks and mental health can be significantly impacted by social media [28]. The result is consistent with a previous study that perceived vulnerability to COVID-19 mediated the relationship between media exposure and depressive symptoms [67]. Exceeding information and communication about COVID-19 on social media reinforces feelings of risk and higher levels of risk perception increase university students’ depressive symptoms. Imai et al. examined the relationship between risk perception and depression among older adults and found that there was no significant correlation [68]. However, the current study confirmed the association among university students. A possible explanation may be that the affordability of health-related risks increases with age. This finding combines the information mechanism and response mechanism of risk, expanding the social amplification framework of risk. As an amplification station, social media, affects individuals’ risk perception through the processing and dissemination of information, thus forming a response mechanism to psychological health.

Besides, social media fatigue also mediated the relationship between social media overload and depressive symptoms, supporting hypotheses H3a and H3b. It has been confirmed that information overload and communication overload were positively associated with social media fatigue. And there is a positive relationship between social media fatigue and internalizing symptoms such as depression and anxiety [69]. Fatigue was highly associated with psychological factors such as depression and anxiety [70]. In addition, it has also been found that individuals with depressive symptoms are more likely to experience social media fatigue. Being bombed by various kinds of information and communications from social media may lead to fatigue and disrupted task processing, impairing self-regulation and emotional control [71]. This finding provides empirical evidence that social media fatigue may be the antecedent of depressive symptoms. Based on the stressor-strain-outcome (SSO) framework, the present study explored a conceptual research model to show how two different stressors, namely, information overload and communication overload, affect people’s psychological states under the COVID-19 infodemic.

The study yielded interesting findings that risk perception of COVID-19 and social media fatigue played a chain mediating role in the relationship between communication overload and depressive symptoms, lending support to hypothesis H4b. However, this chain-mediated relationship did not make sense between information overload and depressive symptoms, and hypothesis H4a has not been supported. Information overload focuses more on the processing of a huge amount of information that people seek. Pariser proposed the concept of “bubble filters.” The search algorithm pushes personalized information that conforms to the original attitude to individuals through user preferences, which leads to increasingly narrow views on available information, this narrowing creates an invisible filter bubble [72]. The information based on personal preferences (such as the pandemic risk and negative information) affects the level of risk perception. People may turn to social media for further information seeking the effect of the high level of risk perception [73].

Communication overload is concerned with interruptions and frequent unplanned communication that is initiated by a third party [74]. Communication overload is related to both passive and active usage including racking, receiving, and sending [75]. Due to the uncontrollability of communication overload, the risk perception affected by communication overload may lead to social media fatigue. Therefore, risk perception of COVID-19 and social media fatigue played a chain-mediating role in the association between communication overload and depressive symptoms.

Chinese government adopted a zero-COVID strategy and took strict lockdown measures during the COVID-19 [76]. Multiple studies have shown that strict lockdown policies increase people’s psychological burden [76, 77]. The present study provides experimental evidence for the psychological health status of college students in this context. Therefore, in the face of future public health emergencies, protecting people’s mental health issues should become an important consideration for policymakers.

There are some limitations of this study. First, this study is a cross-sectional survey, so that the causative interpretations cannot be determined. Besides, we were unable to establish the necessary temporality to assess the incidence of depressive symptoms and rule out reverse causation. Second, this study relied on self-report scales for the measurement of key variables, which may have resulted in response bias due to errors in respondents’ understanding of scale items, false responses, cognitive level, emotional state, etc. Third, there is still a lack of in-depth exploration on the impact of the type of social media (instant communication tools or short videos), the source of information (We media information or official information), and with whom to communicate (families or peers) on depressive symptoms. Fourth, the current study examines university students during the COVID-19 school lockdown, further exploration is needed to determine whether these findings are applicable to other populations.

### Conclusion

This study provides a new perspective for us to understand the impact of social media overload on mental health during public health emergencies. The findings showed that both information overload and communication overload were predictors of depressive symptoms, besides, risk perception of COVID-19 and social media fatigue mediated this relationship. The higher the degree of information overload and communication overload, the stronger people’s risk perception and social media fatigue, thereby increasing the degree of depressive symptoms. We also conducted a chain mediating relationship between communication overload and depressive symptoms. These findings indicate that strict lockdown policies in epidemics can have an impact on the mental health of college students, and the use of social media plays an important predictive role. The present manuscript sparked further thinking on how people should correctly use social media for risk communication under the infodemic. Individuals should reduce the discussion and dissemination of uncertain information during public health emergencies, avoiding social media overload and mental health problems. Besides, the findings provide strong evidence for policy-making in public health emergencies. Future public health policies need to be optimized in the following areas: firstly, for government departments, it’s important to make information available, dispel rumors, and create efficient channels for public feedback. Secondly, social media platforms should establish information filtering mechanisms to provide reliable information during public health emergencies and avoid infodemic. Thirdly, public health policymakers should take more into account individual mental health, especially for vulnerable groups like college students. Measures such as providing psychological counseling services should be taken to alleviate the psychological problems of vulnerable groups in public health emergencies.

## Appendix A

**TABLE A1:** Measurement scales for variables.

Information overload	1. I Am often distracted by the excessive amount of information about COVID-19 available to me on social media
	2. I find that I am overwhelmed by the amount of information about COVID-19 I have to process daily on social media
	3. There is too much information about COVID-19 that come from my friends on social media which is hard to handle
	4. I find that only a small part of the information about COVID-19 on social media is relevant to my needs
Communication overload	1. I received too much content about COVID-19 on social media (e.g., pictures, articles, small videos, documents, etc.)
	2. I sent more outbreak-related messages about COVID-19 on social media than I would
	3. I received too many voices, video chats, online meetings and notifications about COVID-19 on social media
	4. I received so many outbreak-related messages about COVID-19 on social media that I missed or forgot to address them
Risk perception of COVID-19	1. How much do you think COVID-19 has impacted society?
	2. What do you think is the threat of COVID-19 to your life and health?
	3. What do you think is the negative impact of COVID-19 on your life and your family’s lives?
	4. How has COVID-19 impacted your work or study with your family?
Social media fatigue	1. Activities that require the use of social media leave me feeling exhausted
	2. Using social media makes me feel exhausted
	3. Using social media is a burden for me
Depressive symptoms	1. I have felt lonely
	2. I did not enjoy my life
	3. I have felt myself unworthy
	4. I have felt all the joy had disappeared from my life
	5. I have felt my sadness was not relieved even with the help of family/friends
